# Data on pigments and long-chain fatty compounds identified in *Dietzia* sp. A14101 grown on simple and complex hydrocarbons

**DOI:** 10.1016/j.dib.2015.07.022

**Published:** 2015-07-29

**Authors:** Ina Hvidsten, Svein Are Mjøs, Gunhild Bødtker, Tanja Barth

**Affiliations:** a Department of Chemistry, University of Bergen, Allégaten 41, Bergen 5007, Norway; b Uni Research CIPR, Uni Research, PO Box 7810, Bergen 5020, Norway

## Abstract

This data article provides:

1. An overview of tentatively identified long chain compounds in *Dietzia* sp. A14101 grown on simple and complex hydrocarbons;
2. Preliminary Identification of pigments in bacterial material obtained from incubation with a hydrocarbon (dodecane, n-C_12_) as the only carbon and energy source;
3. Some pictures to illustrate the cell surface charge test.

**Specifications Table:** 

Subject area	Chemistry, Biology
More specific subject area	Bacterial fatty acids, bacterial pigments, long-chain fatty acids
Type of data	Table, graph, figure
How data was acquired	HPLC instrument: DIONEX ultimate 3000 diode array detector, DIONEX ultimate 3000 autosampler column compartment, DIONEX P680 HPLC Pump (Thermo Fisher Scientific, Waltham, MA, USA); Chromeleon 7 software.
Data format	Analysed
Experimental factors	Incubation: hydrocarbon versus non-hydrocarbon substrate; simple versus complex (mixture) substrate; Sampling of bacterial cells: centrifugation versus microfiltration; pre-treatment: lyophilised versus intact biomass
Experimental features	Long-chain fatty acids tentatively identified by GS–MS. Pigments analysed by TLC and RP-HPLC. Some illustrations of the cell-surface charge test
Data source location	Bergen, Norway
Data accessibility	The data is with this article

**Value of the data**

• Fatty acid composition of this recently discovered Gram-positive strain can be useful for further taxonomical studies of members of this genus.
• Identification of pigments in general and pigments in bacteria in particular (here, in a hydrocarbon-degrading strain) could be of interest for pharmaceutical and food-industry research groups.
• Correlation between physical properties of the cellular membrane and lipid content is important for understanding how prokaryotes interact with diverse surfaces and how such interactions could be enhanced or eliminated.

## 1 Data

The aerobic chemoorganotrophic Gram-positive high G+C hydrocarbon-degrading mesophilic *Dietzia* sp. A 14101 was incubated on a range of media and substrates. The data presented here provides information on (1) the changes observed in the production of the long-chain compounds and (2) pigments produced by *Dietzia* sp. A 14101. Changes in the type and relative amount of the tentatively identified long-chain compounds are subtle. The production of pigments is coupled to growth on the water-immiscible hydrocarbon substrates. Some selected pictures illustrating identification of the cell-surface charge test of the bacterial membrane of *Dietzia* sp. A 14101 are included. The test is time-effective, requires only common laboratory equipment and fresh intact bacterial cells.

## 2 Experimental design, materials and methods

### 2.1 Experimental design

The strain *Dietzia* sp. A 14101 was isolated from an oil reservoir model column ([1]). In total nine incubations on a range of media (six liquid media and three solid media) and substrates (water-soluble versus water-immiscible) were performed ([4]). In short, centrifugation (5000×*g*; 2×20 min) and microfiltration (successively through 1.2 μm, 0.8 μm and 0.2 μm polycarbonate filters) were employed for separating biomass from liquid media. Biomass grown on solid media was sampled by scraping with a spatula. The obtained cell material was spitted into replicates. Further, selected replicates were lyophilized while the rest of the crude biomass was analyzed as it was.

### 2.2 Materials

Solvents, all HPLC and p.a. grade, and chemicals (sodium azide, NaN_3_; butylated hydroxytoluene, BHT), pigment standards (β-carotene and lycopene) were purchased from Sigma-Aldrich (Oslo, Norway).

Thin layer chromatographic plates (MACHEREY_NAGEL, DC-Fertgfolien ALUGRAM® SIL G/UV254, silica gel 60 with fluorescent indicator; layer thickness 0.20 mm), TLC saturation pads (Analtech 8124, size 20×20 cm, pkg of 100 pieces), micro-pipettes for the application of samples (Drummond) and a nebuliser (10 ml) were bought from Sigma-Aldrich (Oslo, Norway).

The crude oil (non-biodegraded, The Statfjord oil field, the Norwegian continental shelf) was filtered (0.2 μm) and autoclaved.

The methylation reagent, dry HCl in methanol (2.5 M) was prepared as described by [5].

### 2.3 Methods

1. Whole-cell acidic methanolisis was performed as described by [5]. In short, the material was directly methylated in a derivatisation tube in the appropriate amount of the reagent. Iso-octane was used for extraction and the concentration of the extracts was adjusted for GC–MS.

The GC–MS system consisted of a Trace GC Ultra coupled to a DSQ II quadrupole mass spectrometer (Thermo Fisher Scientific, Waltham, MA, USA). The capillary column was a BPX70 (SGE, Ringwood, Australia) with 60 m length, internal diameter of 0.25 mm and a film thickness of 0.25 μm. The program conditions were as described by [4]. Analysis of GC–MS data was performed in Chrombox Q 12-01 (www.chrombox.orgwww.chrombox.org) using libraries for the BPX70 column available at www.chrombox.org/datawww.chrombox.org/data. The methodology applies retention indices combined with mass spectra for reliable identification of compounds as described in Wasta and Mjøs [11].

2. Experimental conditions and methods employed for the preliminary identification of pigments by Thin Layer Chromatography (TLC) and Reverse Phase High Performance Liquid Chromatography (RP-HPLC) were as follows:

Pigments were extracted according to [7].

The TLC elution system employed was toluene—chloroform-methanol, (85:15:5, by vol. %).

Reverse phase high performance liquid chromatography gradient method was used to separate species in the carotenoid extracts. The following analytical conditions used (by courtesy of Prof. T. Fossen) a C_18_ column (ZORBAX SB-C18, 5 μm, 4.6×250 mm, Agilent Technologies, USA) and a UV/Vis multiple channel detection (*λ*_max_ 470±16 nm non-polar; 450±16 nm polar carotenoids), with the following solvent programme: 0–25 min. 10% methanol, 85% acetonitrile, 5% hexane-dichloromethane (1:1, by vol.); 26–45 min. 10% methanol, 65% acetonitrile, 25% hexane-dichloromethane (1:1, by vol.); 46–60 min. 10% methanol, 85% acetonitrile, 5% hexane-dichloromethane (1:1, by vol.).

3. Cell-surface charge test was performed as described by Wang and Langley [10] and modified by [3].

### 2.4 Data

#### 2.4.1 Long chain compounds

**Fig. 1:**
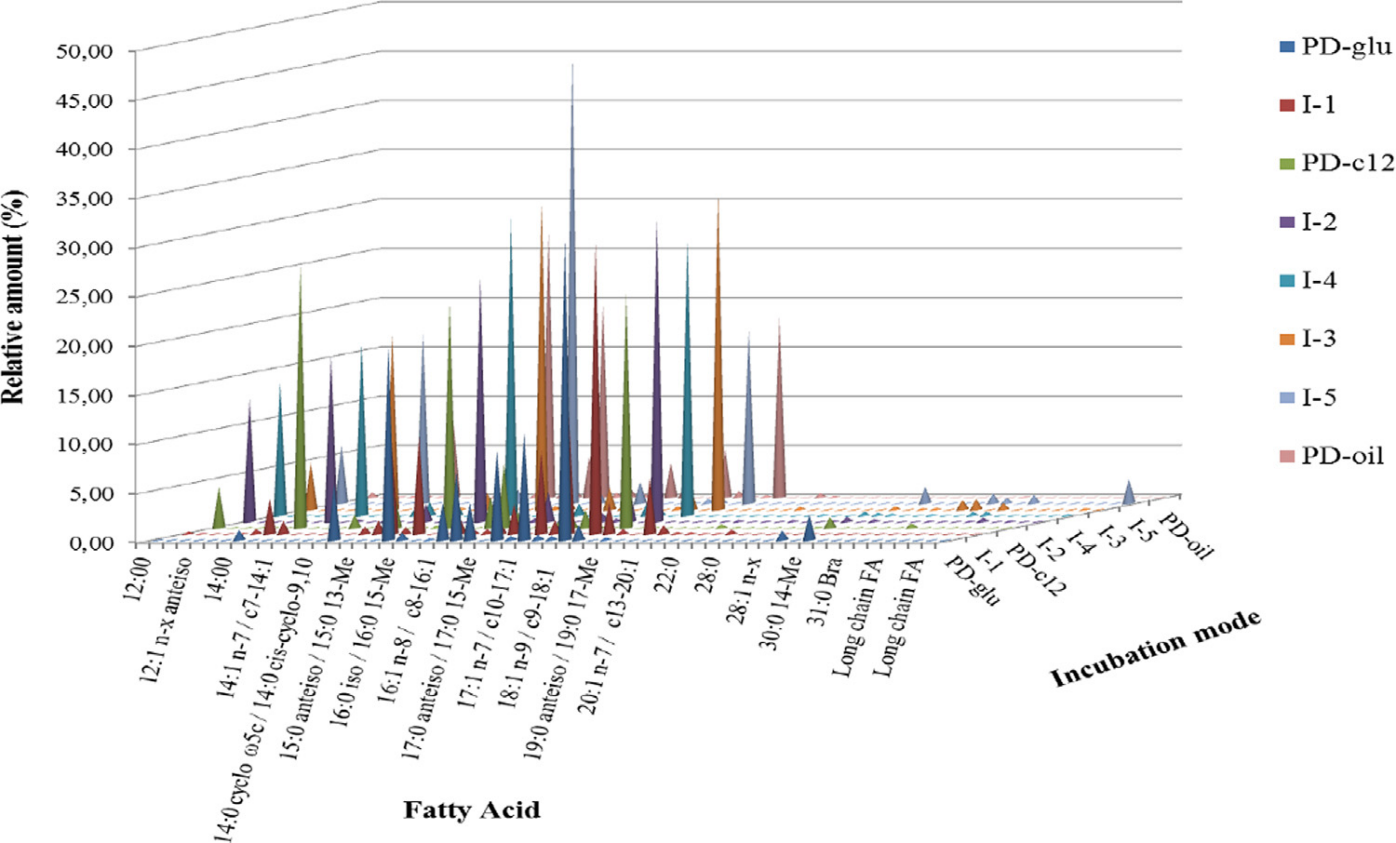
The 3D profile of FA, for the range of “C_12:0_–C_28:0_ plus” produced by *Dietzia* sp.A14101 during the growth on different media and diverse substrates. The diagram is rotated so that the region of the tentatively identified long-chain structures (fatty acids including tentatively identified long-chain fatty compounds and/or mycolic acids) is clearly displayed.

Along with the unambiguously identified FA, several long-chain structures were present in the samples obtained from both non- and -lyophilised crude cell material by direct whole-cell acidic methanolisis with the subsequent GC–MS analysis. The overall profile of the separated fatty acid methyl ester was dominated by short chain (less than 22 carbon-atom long chains) FA, see Fig. 1. The relative percent content of the detected species is summarised in Table 1.

The examination of MS spectra of the late eluting compounds in GC–MS chromatograms revealed several long chain compounds (1) aldehydes (mostly saturated) and (2) long-chain branched compounds. The MS spectra were typical for lower molecular weight mycolic acids, MA, defined roughly as having less than 35 carbon atoms, (the fragmentation pattern of MA is discussed in e.g. [2,9]. The examination of spectra suggested that short chain MA, with up to 35 carbon atoms in the parent MA, were present. However, the content varied greatly both qualitatively and semi-quantitatively (several homologues were produced at trace amounts), Table 1. The side-chain composition varied along with the substrate. For incubation I-1 majority species had ECL index range 25 to 27; the main side-chain was odd-numbered and was 13 or 15 carbon atoms long, while for the incubation PD-glu the main side chains were 8, 10, or 12 carbon atoms long and the ECL index range was 27–28. The data indicated that for all incubations on the simple HC-substrate side chain length was C8, C10, C12, C14 and C15. For PD-oil ECL index range was 23–24 and above 28, the dominant side-chain lengths were 14 or 16 carbon atoms long. No indication of other than a 3-hydroxy acid unit functions was found. Mass spectra indicated the presence of a single double bond in several tentatively identified MA in incubation I-1, and a spectrum of only one homologue indicated presence of two double bonds. Thus, the tentatively identified short-chain mycolic acids (SCMA) truly belong to the subgroup of α- and α′- mycolic acids according to [6]. The MS spectra also indicated the both odd- and even number side-chains were produced by *Dietzia* sp. A14101 independent of the substrate or media. Similar data were reported for D. maris 58001^T^ ([8]), who further suggested that this could be result of a novel pathway of fatty acid biosynthesis. Lyophilised samples produced more consistent and reproducible results, while fresh wet-cell type samples were better suited for qualitative analyses. The glucose substrate gave high amounts of long-chain fatty compounds compared to the rest of the incubations and especially the solid phase PD-oil incubation (Table 2).

#### 2.4.2 Pigments

Pigments in the bacterial material obtained from incubations of *Dietzia* sp. A14101 on a hydrocarbon water-immiscible substrate (dodecane, n-C12) as the only carbon and energy source were analysed by TLC and RP-HPLC.

Material sampled from incubations I-2 (lyophilised) and I-3 and I-5 (“wet” cells) were analysed. Detection was based on TLC *R*_*f*_ —values, RP-HPLC retention times and absorption wavelengths.

**Fig. 2:**
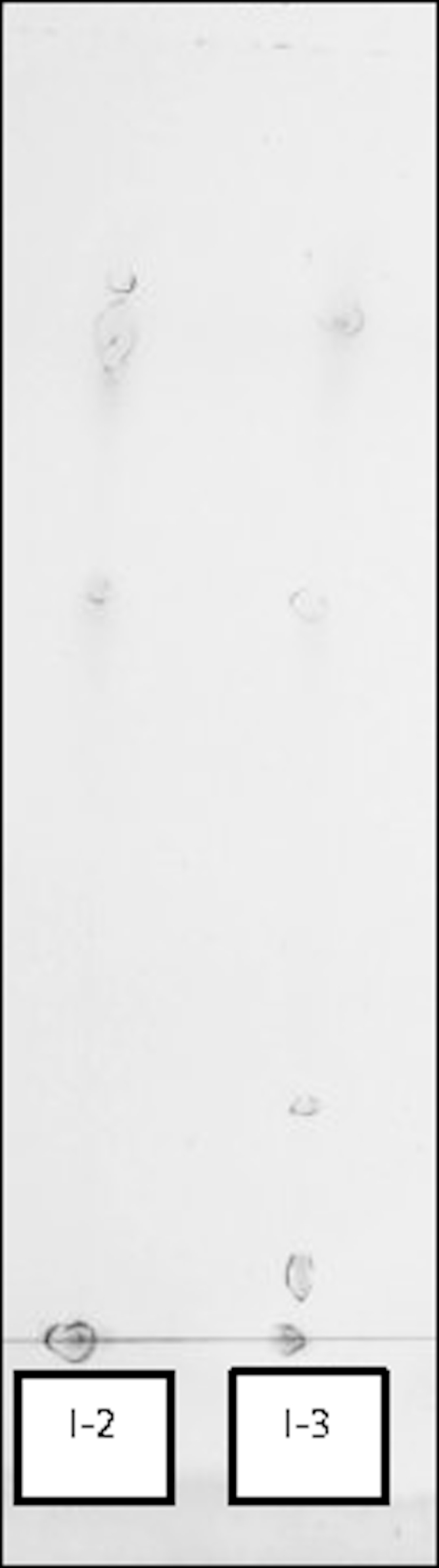
An example of a developed TLC chromatogram; the solvent front form the origin was 9 cm.

**Fig. 3:**
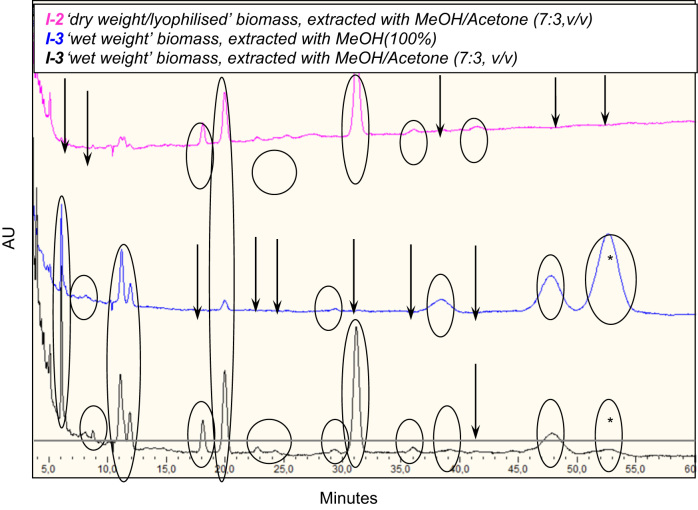
A comparative HPLC chromatogram: lyophilised (I-2, pink) versus “wet cells” (I-3, blue and black) cell extracts. The arrows indicate the peaks lost either due to pre-treatment by lyophilisation or due to the extraction with only a polar solvent (here, methanol). * Lycopene. (For interpretation of the references to color in this figure legend, the reader is referred to the web version of this article.)

TLC provided very rough separation of the pigments present and confirmed that lycopene was the dominant non-polar pigment (*R*_*f*_ 0.8) produced. Both TLC and RP-HPLC analyses suggested presence of polar pigments of the astaxanthin type (*R*_*f*_ 0, 0.07, 0.4, 0.54), Fig. 2 and Table 3, and RP-HPLC, Fig. 3. The further identification was not pursued further. The preliminary identification of pigments suggested that lyophilized samples (Incubation-2) gave poor extraction yields, Fig. 2, of pigments if compared to “wet” cell material (e.g. Incubation-3). Proper separation and accurate quantification of the astaxanthin isomers requires de-esterification of the pigment(s) that is/are otherwise bound/esterified with fatty acids.

#### 2.4.3 Some pictures illustrating the cell surface charge test

**Fig. 4:**
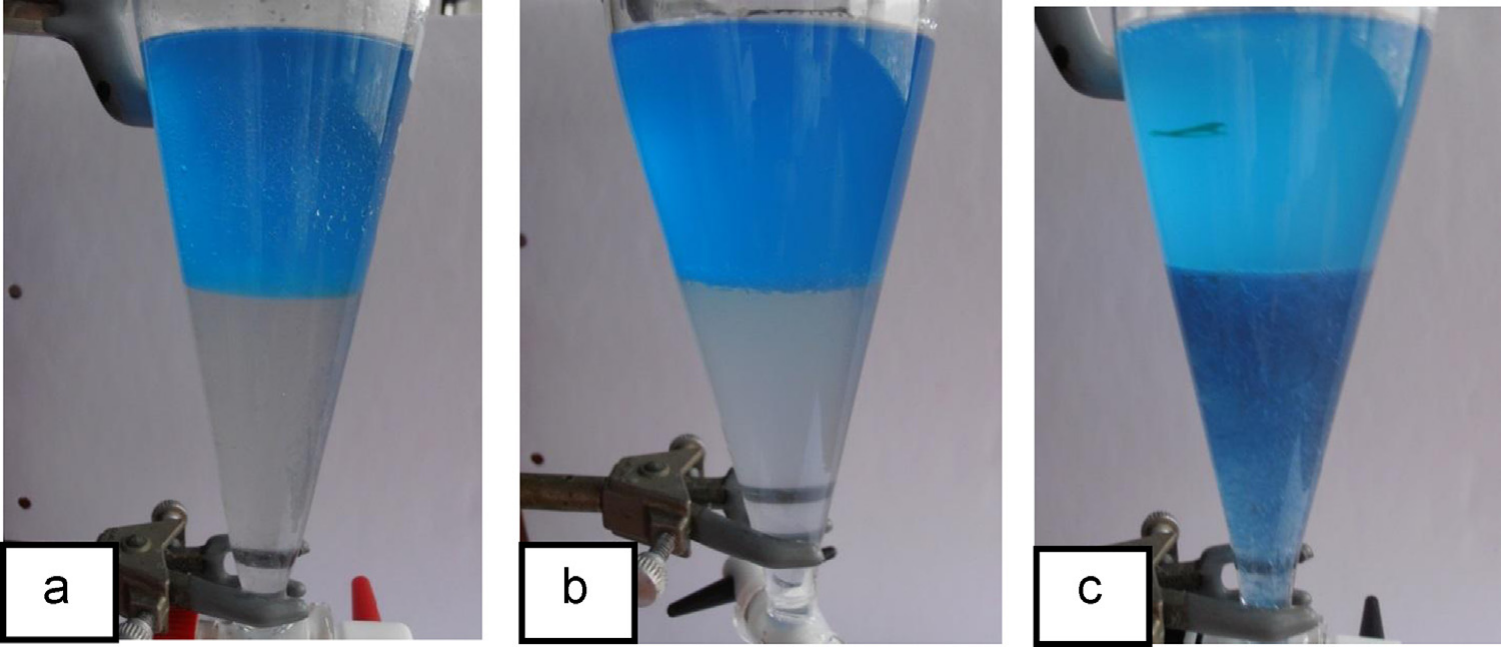
(a) Cell surface charge for cells (1.5 g) grown on glucose—clear CHCl_3_ phase (the lower phase); (b) Cell surface charge for cells grown on crude oil vapour—weakly blue CHCl_3_ phase (the lower phase), a small amount of cells (0.5 g) was used for this test; (c) Cell surface charge for cells (1.5 g) grown on crude oil vapour—strong blue CHCl_3_ (the lower phase). (For interpretation of the references to color in this figure legend, the reader is referred to the web version of this article.)

Fig. 4(a–c) illustrate the test results obtained for cells cultured on different substrates on Petri Dish. The test was performed on the cells collected by centrifugation (thus retaining substantial amounts of n-C_12_), the cells collected by microfiltration (retaining no n-C_12_) and the cells grown on the hydrocarbon substrate vapour (thus free from n-C_12_). In the case of solid medium Petri dish incubation on dodecane (PD-c12) and solid medium Petri dish incubation on glucose (PD-glu) the whole Petri Dish cell cultures were used. In case of solid medium incubation on crude oil as a substrate (PD-oil) only one well-defined colony cluster was collected for this analysis.

## Figures and Tables

**Table 1:** The profiles of the tentatively identified long-chain fatty acids produced by *Dietzia* sp. A14101 during the growth on non- and HC-substrates. Values are percentages of total fatty acids with respect to the amount and type of the material used. **n.d.**=not detected; **I**=incubation; **PD**=Petri dish.

**Incubation reference name**	**PD-glu**	**I-1**	**PD-c12**	**I-2**	**I-4**	**I-3**	**I-5**	**PD-oil**
**FA**	**%**	**%**	**%**	**%**	**%**	**%**	**%**	**%**

28:0 (Branched, 14:0)	n.d.	0.07	n.d.	0.20	0.43	0.34	1.71	n.d.
28:1 *n*–*x*	n.d.	0.08	0.10	0.56	0.32	0.01	n.d.	n.d.
28:1 *n*–*x*	n.d.	n.d.	1.15	0.06	0.25	0.18	n.d.	n.d.
29:0/1 *n*–*x* (Branched-anteiso,14:0)	1.03	n.d.	n.d.	0.35	n.d.	0.21	n.d.	n.d.
28:1 *n*–*x*	n.d.	n.d.	n.d.	n.d.	n.d.	n.d.	n.d.	n.d.
30:0 14-Me	2.65	0.12	n.d.	n.d.	0.22	1.01	1.06	0.13
30:0 12-Me	n.d.	n.d.	n.d.	0.05	n.d.	1.10	0.59	n.d.
c9-30:0 12-Me	n.d.	n.d.	n.d.	0.12	n.d.	n.d.	n.d.	n.d.
31:1 *n*–*x* Branched	n.d.	n.d.	0.51	0.08	0.39	0.71	0.90	n.d.
31:1 *n*–*x* Branched	n.d.	n.d.	n.d.	n.d.	0.43	n.d.	n.d.	n.d.
31:1 *n*–*x*	n.d.	n.d.	n.d.	0.11	n.d.	n.d.	0.02	n.d.
32:1 *n*–*x*	n.d.	n.d.	n.d.	0.02	0.21	0.01	2.51	0.06
Long chain FA	n.d.	n.d.	n.d.	0.48	n.d.	n.d.	n.d.	n.d.
Long chain FA	n.d.	n.d.	n.d.	0.02	n.d.	n.d.	n.d.	n.d.
Long chain MA	n.d.	n.d.	n.d.	0.17	n.d.	n.d.	n.d.	n.d.
Long chain FA	n.d.	n.d.	n.d.	0.04	n.d.	0.19	n.d.	n.d.

Total	3.68	0.27	1.76	2.26	2.25	3.76	6.79	0.19

**Table 2:** A short summary of the observed changes in the relative content of long-chain fatty acids as a function of the substrate.

**Incubation reference name**	**Medium**	**Substrate**	**Relative content (long-chain FA incl. MA)**	**Type and the amount crude pellet per extraction (g)**
**I-1**	Nutrient rich, liquid	Non-HC	Low content, few species.	1.5 (lyophilised)
**PD-glu**	Solid	Non-HC	Low content, few species.	≈0.5 (wet)
**I-2**	Defined medium, liquid	HC, simple	High content, wide range of species.	1 (lyophilised)
**I-3**				1.5 (wet)
**I-4**				1 (lyophilised)
**I-4**				1.5 (wet)
**PD-c12**	Solid	HC, simple	Low content, few species	≈0.5 (wet)
**PD-oil**	Solid	HC, mixture	Low content, few species	≈0.7 (wet)

**Table 3:** The calculated *R*_*f*_*-*values for the TLC spots; the solvent front form the origin was 9 cm.

**Spot**	**Migration distance (cm)**	***R***_***f***_**=distance**_**from origin**_**distance**_**solvent front**_	**Identity**
5	7.3	0.81	Lycopene
4	6.2	0.69	Unknown pigment
3	4.9	0.54	Unknown pigment
2	3.6	0.40	Polar pigment (s)
1	0.7	0.08	Polar pigment(s)
*i*	Origin	0.00	Polar pigment(s)
