# Supplementary material for: Data on pigments and long-chain fatty compounds identified in Dietzia sp. A14101 grown on simple and complex hydrocarbons
Source: Data Brief. 2015 Jul 29;4:622–9. doi: 10.1016/j.dib.2015.07.022 (PMC4552950; doi:10.1016/j.dib.2015.07.022)
Supplement: Supplementary file 1 — Supplementary data [file mmc1.zip › Source File Suppl Table 1 Hvidsten.docx]

Source File

Data in Brief, Table 1.

| **Incubation reference name** | **PD-glu** | **I-1** | **PD-c12** | **I-2** | **I-4** | **I-3** | **I-5** | **PD-oil** |
| --- | --- | --- | --- | --- | --- | --- | --- | --- |
| **FA** | ***%*** | ***%*** | ***%*** | ***%*** | ***%*** | ***%*** | ***%*** | ***%*** |
|  |  |  |  |  |  |  |  |  |
| 28:0 (Branched, 14:0) | *n.d.* | 0,07 | *n.d.* | 0,20 | 0,43 | 0,34 | 1,71 | *n.d.* |
| 28:1 n-x | *n.d.* | 0,08 | 0,10 | 0,56 | 0,32 | 0,01 | *n.d.* | *n.d.* |
| 28:1 n-x | *n.d.* | *n.d.* | 1,15 | 0,06 | 0,25 | 0,18 | *n.d.* | *n.d.* |
| 29:0/1 n-x (Branched-anteiso,14:0) | 1,03 | *n.d.* | *n.d.* | 0,35 | *n.d.* | 0,21 | *n.d.* | *n.d.* |
| 28:1 n-x | *n.d.* | *n.d.* | *n.d.* | *n.d.* | *n.d.* | *n.d*. | *n.d.* | *n.d.* |
| 30:0 14-Me | 2,65 | 0,12 | *n.d.* | *n.d.* | 0,22 | 1,01 | 1,06 | 0,13 |
| 30:0 12-Me | *n.d.* | *n.d.* | *n.d.* | 0,05 | *n.d.* | 1,10 | 0,59 | *n.d.* |
| c9-30:0 12-Me | *n.d.* | *n.d.* | *n.d.* | 0,12 | *n.d.* | *n.d.* | *n.d.* | *n.d.* |
| 31:1 n-x Branched | *n.d.* | *n.d.* | 0,51 | 0,08 | 0,39 | 0,71 | 0,90 | *n.d.* |
| 31:1 n-x Branched | *n.d.* | *n.d.* | *n.d.* | 0,00 | 0,43 | *n.d.* | *n.d.* | *n.d.* |
| 31:1 n-x | *n.d.* | *n.d.* | *n.d.* | 0,11 | *n.d.* | *n.d.* | 0,02 | *n.d.* |
| Long chain FA | *n.d.* | *n.d.* | *n.d.* | 0,48 | *n.d.* | *n.d.* | *n.d.* | *n.d.* |
| Long chain FA | *n.d.* | *n.d.* | *n.d.* | 0,02 | *n.d.* | *n.d.* | *n.d.* | *n.d.* |
| Long chain MA | *n.d.* | *n.d.* | *n.d.* | 0,17 | *n.d.* | *n.d.* | *n.d.* | *n.d.* |
| Long chain FA | *n.d.* | *n.d.* | *n.d.* | 0,04 | *n.d.* | 0,19 | *n.d.* | *n.d.* |
| 32:1 n-x | *n.d.* | *n.d.* | *n.d.* | 0,02 | 0,21 | 0,01 | 2,51 | 0,06 |
