# Supplementary material for: Data on pigments and long-chain fatty compounds identified in Dietzia sp. A14101 grown on simple and complex hydrocarbons
Source: Data Brief. 2015 Jul 29;4:622–9. doi: 10.1016/j.dib.2015.07.022 (PMC4552950; doi:10.1016/j.dib.2015.07.022)
Supplement: Supplementary file 1 — Supplementary data [file mmc1.zip › Source File Suppl Table 2 Hvidsten.docx]

Source File

Data in Brief, Table 2.

| **Incubation reference name** | **Medium** | **Substrate** | **Relative Content (long-chain FA incl. MA)** | **Type and the amount crude pellet per extraction (g)** |
| --- | --- | --- | --- | --- |
| **I-1** | Nutrient rich, liquid | Non-HC | Low content, few species. | 1.5 (lyophilised) |
| ***PD-glu*** | Solid | Non-HC | Low content, few species. | ≈ 0.5 (wet) |
| **I-2** | Defined medium, liquid | HC, simple | High content, wide range of species. | 1 (lyophilised) |
| **I-3** |  |  |  | 1.5 (wet) |
| **I-4** |  |  |  | 1 (lyophilised) |
| **I-4** |  |  |  | 1.5 (wet) |
| ***PD-c12*** | Solid | HC, simple | Low content, few species | ≈ 0.5 (wet) |
| ***PD-oil*** | Solid | HC, mixture | Low content, few species | ≈ 0.7 (wet) |
