# Supplementary material for: Data on pigments and long-chain fatty compounds identified in Dietzia sp. A14101 grown on simple and complex hydrocarbons
Source: Data Brief. 2015 Jul 29;4:622–9. doi: 10.1016/j.dib.2015.07.022 (PMC4552950; doi:10.1016/j.dib.2015.07.022)
Supplement: Supplementary file 1 — Supplementary data [file mmc1.zip › Source File Suppl Table 3 Hvidsten.docx]

Source File

Data in Brief, Table 3.

| **Spot** | **Migration distance**  **(*cm*)** | **R_f_ = distance _from origin_**  **distance _solvent front_** | **Identity** |
| --- | --- | --- | --- |
| 5 | 7,3 | 0,8111 | Lycopene |
| 4 | 6,2 | 0,6889 | Unknown pigment |
| 3 | 4,9 | 0,5444 | Unknown pigment |
| 2 | 3,6 | 0,4 | Polar pigment (s) |
| 1 | 0,7 | 0,0778 | Polar pigment(s) |
| *i* | origin | 0 | Polar pigment(s) |
