# Supplementary figures and images for: Data on pigments and long-chain fatty compounds identified in Dietzia sp. A14101 grown on simple and complex hydrocarbons
Source: Data Brief. 2015 Jul 29;4:622–9. doi: 10.1016/j.dib.2015.07.022 (PMC4552950; doi:10.1016/j.dib.2015.07.022)

Source File

Data in Brief, Figure 4 (a-b-c).


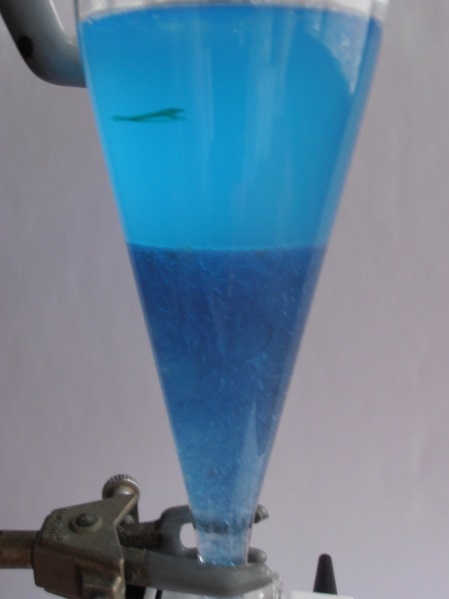

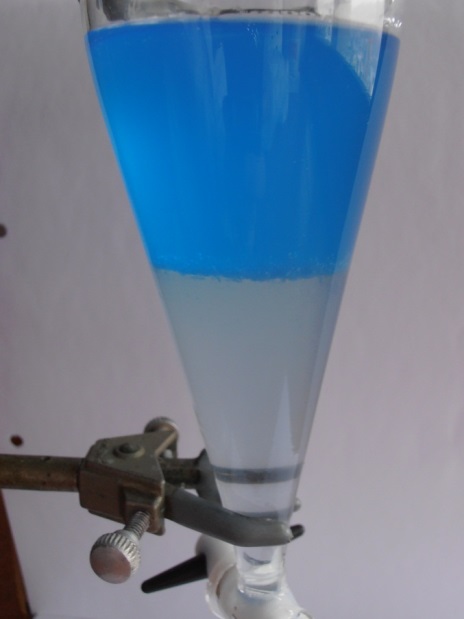

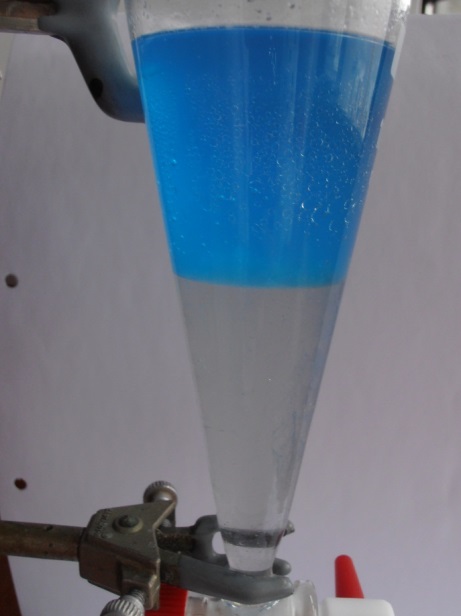


(c)

(b)

(a)

Supplement: Supplementary file 1 — Supplementary data [file mmc1.zip › Source File Suppl Fig 4a-b-c Hvidsten.docx]

Source File

Data in Brief, Figure 1.

**Picture:**

**
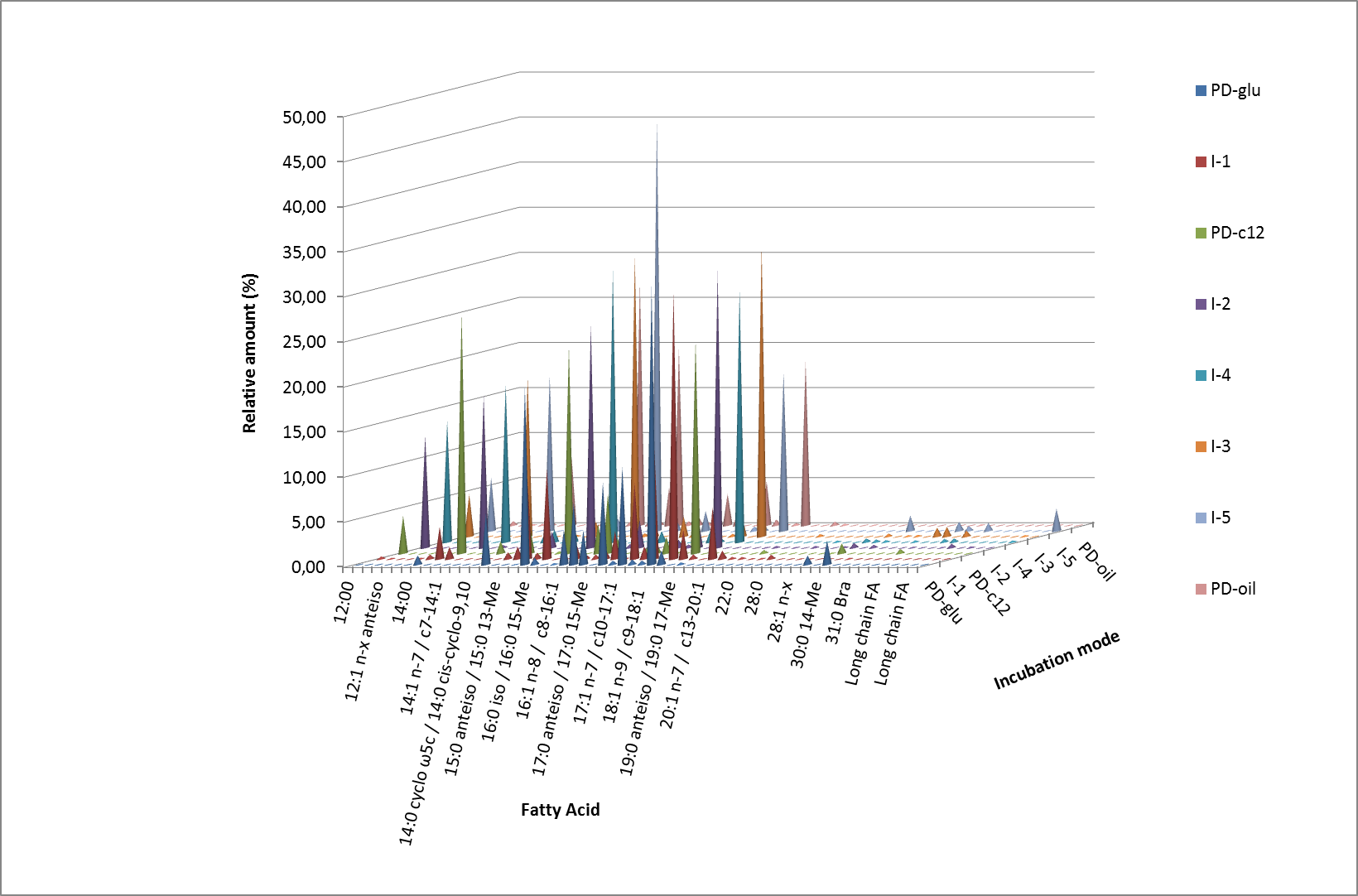
**

**Kept with Source Formatting and Embedded Workbook**:

Supplement: Supplementary file 1 — Supplementary data [file mmc1.zip › Source File Suppl Fig 1 Hvidsten.docx]

Source File

Data in Brief, Figure 2.


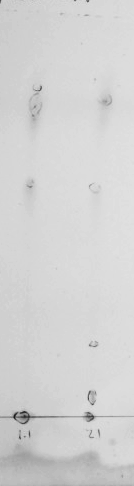


I-3

I-2

Supplement: Supplementary file 1 — Supplementary data [file mmc1.zip › Source File Suppl Fig 2 Hvidsten.docx]

Source File

Data in Brief, Figure 3.

**I-3** 'wet weight' biomass, extracted with MeOH/Acetone (7:3, v/v)

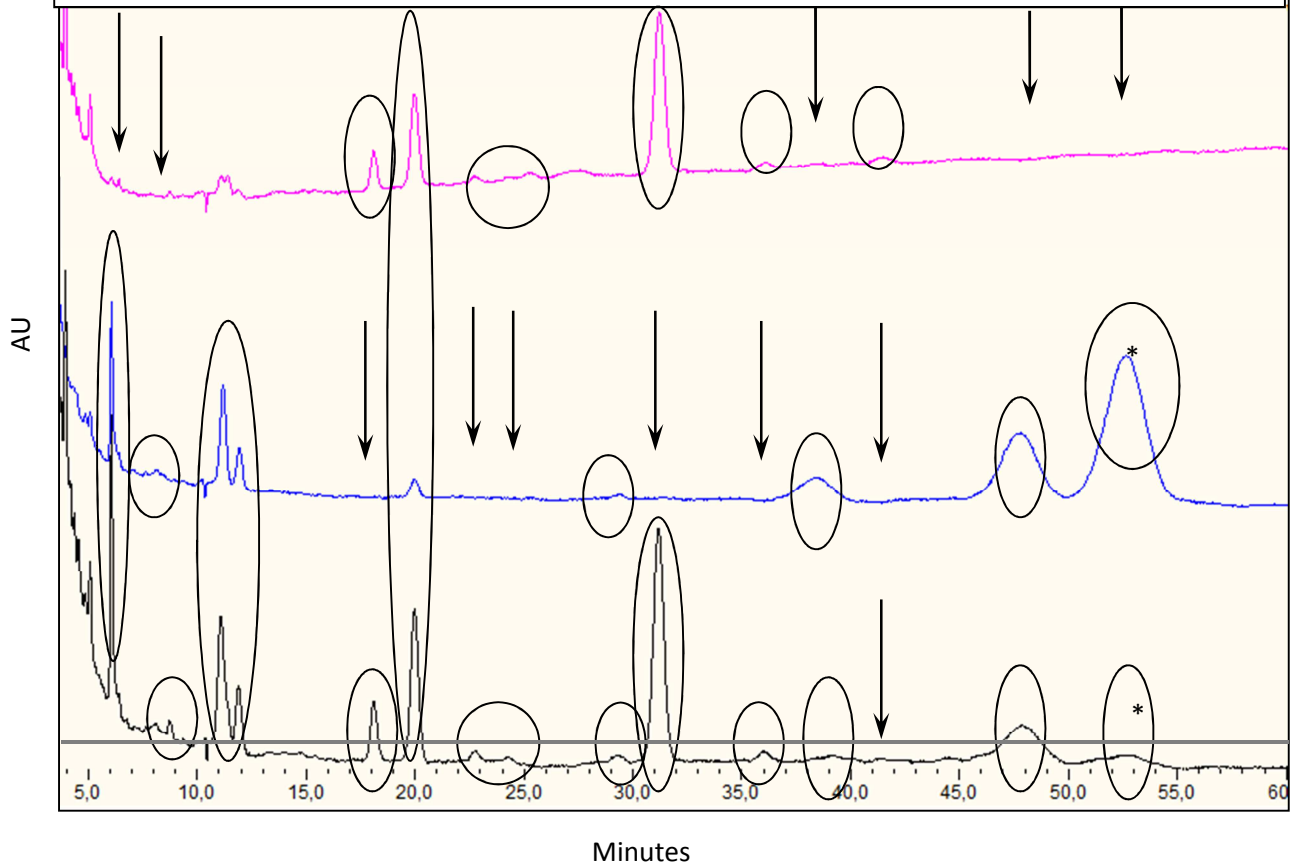

Supplement: Supplementary file 1 — Supplementary data [file mmc1.zip › Source File Suppl Fig 3 Hvidsten.pdf]
